# Maturation Fetus Ascending Aorta Elastic Properties: Circumferential Strain and Longitudinal Strain by Velocity Vector Imaging

**DOI:** 10.3389/fcvm.2022.840494

**Published:** 2022-02-28

**Authors:** Xin Zhong, Yuanchen Luo, Dan Zhou, Minghui Liu, Jiawei Zhou, Ran Xu, Shi Zeng

**Affiliations:** ^1^Department of Ultrasound, Hunan Provincial People's Hospital (The First Affiliated Hospital of Hunan Normal University), Changsha, China; ^2^Department of Ultrasound Diagnosis, The First Hospital of Changsha, Changsha, China; ^3^Department of Ultrasound Diagnosis, The Second Xiangya Hospital, Central South University, Changsha, China; ^4^Department of Urology, The Second Xiangya Hospital, Central South University, Changsha, China

**Keywords:** velocity vector image technique, aortic elasticity mechanics, fetus, strain, speckle tracking technique

## Abstract

**Objective:**

This study aimed to assess the circumferential and longitudinal strain of the fetal ascending aortic (AA) wall and establish a gestational age-associated longitudinal reference for aortic wall strain during the second half of pregnancy.

**Methods:**

Singleton fetuses with gestational age (GA) at 20 + 0 to 24 + 6 weeks were prospectively collected from a low-risk population. Global circumferential strain (GCS) and mean longitudinal strain (MLS) of the ascending aorta were measured serially at 4-week intervals using the velocity vector imaging (VVI) technique. Fractional polynomials were conducted to obtain the best-fitting curves between GA and AA strains. GA-specific reference percentiles of GCS and MLS were established by multilevel modeling.

**Results:**

A total of 223 fetuses with a total of 1,127 serial observations were enrolled. GCS presented a second-degree fractional polynomial smoothing regression along GA (*R*^2^ = 0.635, *P* < 0.05). Fetal aortic GCS remained unchanged at ~27.29% (20.36–35.6%) before 31 weeks and increased significantly from 31.36% (26.38–37.12%) at 31 weeks to 43.29% (30.5–56.78%) at term. MLS presented a third-degree fractional polynomial smoothing regression along GA (*R*^2^ = 0.465, *P* < 0.05). MLS remained steady at ~10.03% (3.28–17.62%) between 20 and 31 weeks and then increased significantly from 12.68% (7.42–20.1%) at 32 weeks to 17.5% (9.67–25.34%) at term. The GCS was significantly higher than the MLS in the ascending aorta wall (*p* < 0.001).

**Conclusion:**

The fetal ascending aorta wall demonstrates obviously greater circumferential strain than longitudinal strain. Both strains remained steady before the late trimester and then gradually increased until delivery, suggesting progressive maturation of aortic elasticity mechanics.

## Introduction

The aorta is a conduit to carry blood away from the heart to the peripheral arteries. More importantly, it exhibits an impedance matching function (1): it can expand in systole after absorbing most of the left ventricular contraction energy and recoil during diastole to guarantee continuous blood flow throughout the cardiac cycle. This buffering effect depends on the well-defined elastic lamella in the vessel wall (2). The elastic lamella, consisting of elastic fibers interspersed with vascular smooth muscle cells (SMCs), provides the essential elastic properties of the aortic wall, which allow the aorta to reversibly stretch and recoil (2). They are formed within a narrow time during humans' lifespan beginning in mid-gestation, maximum accumulation in the perinatal period, further rapid decline, and ending in adolescence (3–6). Owing to the low synthesis of elastin after birth, impaired elastin synthesis during the fetal period may cause decreased compliance of large arteries and even program the development of hypertension and cardiovascular diseases later in life (1, 7). Previous reports on fetal aortic wall mechanics have mostly focused on changes in abdominal aorta intima-media thickness (8–10) and lumen diameter (11, 12) or area changes (13). However, these parameters cannot directly evaluate the mechanical elastic properties of the aortic wall under cyclic stress and pressure.

Velocity vector imaging (VVI), based on a two-dimensional speckle-tracking technique, was originally proposed as a quantitative tool for assessing myocardial deformations (14). It was first applied to assess aortic mechanic characteristics by Kim et al. (15) and was shown to be strongly correlated with pulse wave velocities (PWVs), which is the recommended gold standard measurement of arterial stiffness in adults. However, there are no reports on the reference range of fetal aortic wall elastic characteristics by VVI.

The aims of this study were to assess the circumferential and longitudinal strain of the ascending aortic wall during the second half of pregnancy using VVI and establish a gestational age-associated longitudinal reference for the aortic wall strain.

## Methods

A longitudinal prospective observation was conducted at the Second Xiangya Hospital of Central South University in China between April 2020 and May 2021. Singleton fetuses with gestational age (GA) at 20 + 0 to 24 + 6 weeks were enrolled from the low-risk population. The low-risk population was defined as a pregnant woman who was healthy, had normal results from non-invasive prenatal testing (NIPT) of current gestation, and had no history of abnormal pregnancy such as embryo suspension, fetal malformation, and stillbirth. GA was estimated from the day of the last menstrual period and was confirmed by ultrasound measurement of the crown-rump length during the first trimester. The inclusion criteria were as follows: absence of identical structure and chromosomal defects, absence of placenta insufficiency, absence of oligohydramnios or polyhydramnios, and absence of maternal metabolic and cardiovascular disease. For the longitudinal evaluation of aortic elastic properties, each fetus was scanned serially with a 4-week interval until delivered. Written informed consent was obtained from all parents, and this study was approved by the institutional review board at the Second Xiangya Hospital of Central South University.

Routine obstetrical ultrasound examination was performed by one investigator (ZX) using a Voluson E10 (GE Healthcare Ultrasound, Milwaukee, WI, USA) with a RAB 4–8-D probe. Fetal biometry indices, including biparietal diameter, head circumference, femur length, and abdominal circumference, were obtained and combined to calculate an estimated fetal weight (EFW). The pulsative index (PI) of the umbilical artery (UA) was measured at the free-floating loop of the umbilical cord in the absence of fetal movement.

**Figure 1:**
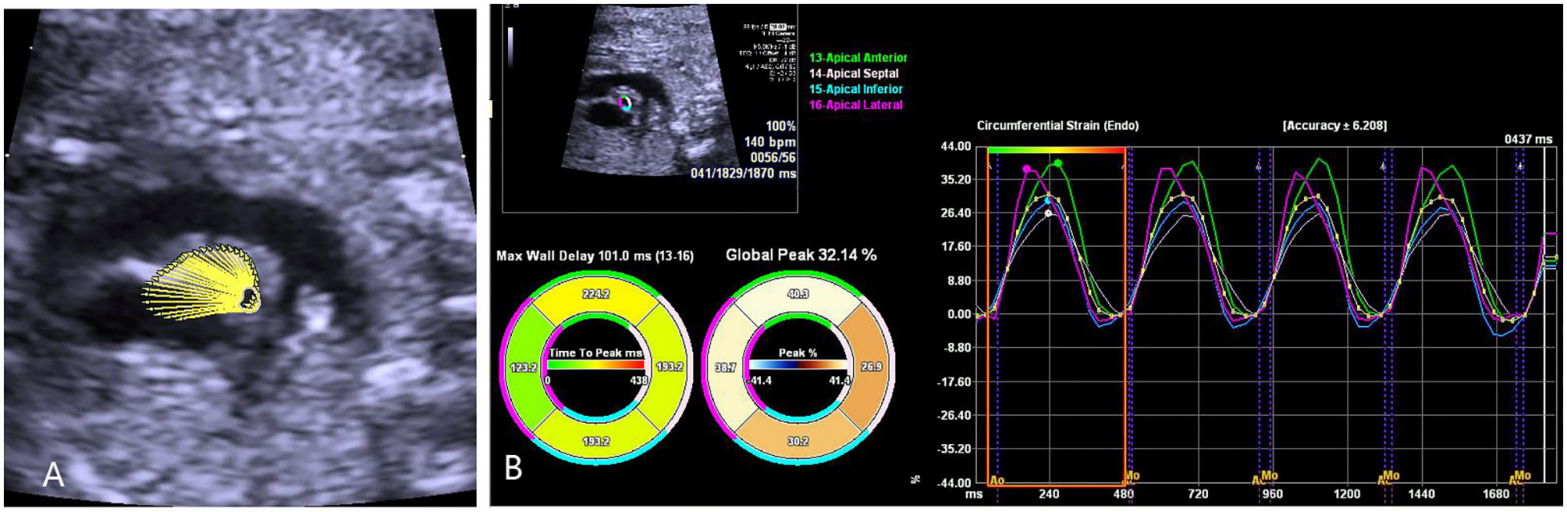
The velocity vector image of the traced fetal ascending aorta is shown in the great artery short-axis view **(A)**. The circumferential strain curve during the cardiac cycle was automatically displayed in the segmental model with a specific color, and global circumferential strain (GCS) was recorded as the average of all segmental systolic peak circumferential strains **(B)**.

**Figure 2:**
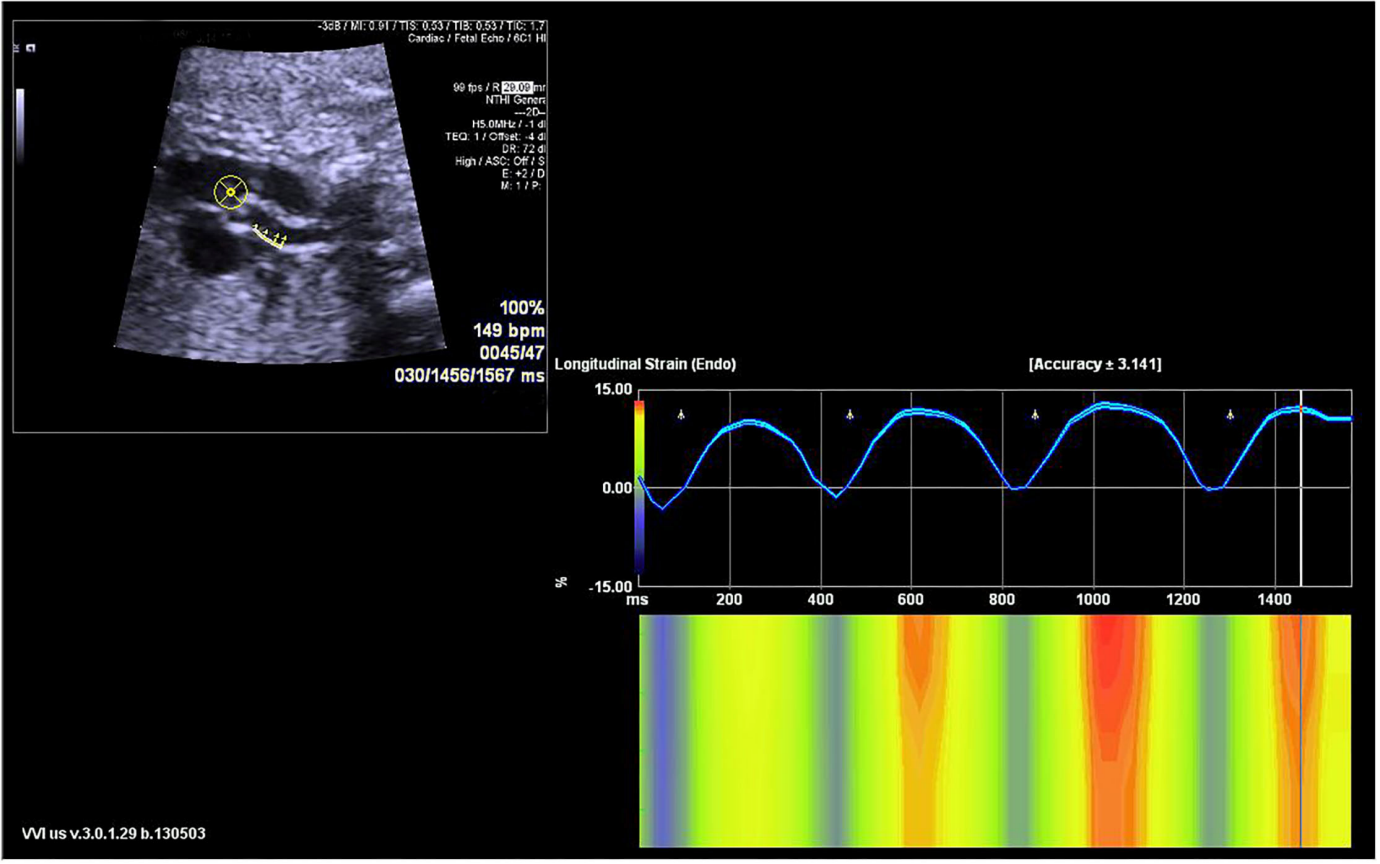
The velocity vector image of the traced posterior ascending aorta wall is shown in the sagittal view **(left upper panel)**. The longitudinal strain curve of the traced wall was automatically calculated and displayed **(right panel)**.

Fetal echocardiography was performed by one expert (ZS) who was blind to the fetal GA using an Acuson SC2000 system (Siemens Medical, WA, USA) with a 6C2 transducer. Standard multiple views of the fetal heart were obtained to evaluate the cardiac anatomy. Ascending aortic (AA) elastic properties were assessed using vector velocity imaging software (VVI; Siemens Medical, Solutions USA, Inc.). Global circumferential strain (GCS) of the ascending aorta was measured in the great artery short-axis view, while the mean longitudinal strain (MLS) of AA was evaluated in the left ventricle outflow tract (LVOT) sagittal view. First, the high-quality cine loop clips (36–54 frames/s) of the abovementioned views with at least 3 heart cycles were acquired and stored. Atrioventricular and semilunar valves needed to be clearly displayed to determine the cardiac phase in the follow-up analysis. Second, annularly tracing the AA along the blood-intima border in a still frame of short-axis view with optimal visualization was performed. The circumferential strain curve during the cardiac cycle was automatically calculated and displayed. GCS was recorded as the average of all segmental systolic peak circumferential strains (Figure 1). Third, the anterior and posterior walls of the AA were traced separately within the course between the sinotubular junction and the roof of the left atrium at the still frame of the LVOT view. Similarly, the longitudinal strain curve of the traced two walls was automatically displayed, and MLS was calculated as the mean systolic peak longitudinal strain of both the anterior and posterior AA walls (Figure 2). AA strains were measured three times, and the mean was used for statistical analyses.

The data were analyzed using STATA 15 (Stata Corp., LLC., College Station, TX, USA). The Shapiro–Wilk *W*-test was used to assess the distribution normality. Fractional polynomials were conducted to obtain the best-fitting curves between GA and AA strains. As in our previous report (16), the establishment of a longitudinal GA-associated reference was as follows. First, box Cox transformation was performed to obtain the appropriate lambda (λ) and normalize the GCS and MLS. Second, multilevel modeling with the restricted maximum likelihood option was performed to obtain the detailed mean and variance of the transformed strains at GA. Finally, the GA-specific reference percentiles were established using Royston P's formulas (17). The difference in AA wall strains among each gestational week was evaluated using one-way ANOVA with *post-hoc* Games-Howell testing. The difference between GCS and MLS was evaluated by paired-samples *t*-test. The Bland-Altman method was performed to test interobserver and intra-observer variability from the stored images of 50 randomly selected observations. Interobserver measurement was performed among ZS and another fetal echocardiographer (XGQ, who was not a co-author), and intra-observer measurement was repeated by ZS in next day. *P* < 0.05 was considered statistically significant.

## Results

The assessment of fetal AA strain was attempted in 238 singleton fetuses. Among which, 15 cases were excluded because of 9 with bad image quality and 7 without follow-up. Finally, 223 fetuses with a total of 1,127 serial observations were enrolled. The GA at the first examination was 22.3 weeks. Each fetus was scanned 4–6 times. The followed time was 16 weeks (12–20 weeks). The flow diagram describing this longitudinal study was shown as Supplementary Figure 1.

The clinical characteristics and outcomes of the cohort are presented in Table 1.

**Table 1:** The clinical characteristics and outcome of the cohort (*n* = 223).

**Parameters**	**Medium (range) or *n* (%)**
**Maternal**	
Age, years	27 (18–41)
BMI, kg/m^2^	23 (18–28.6)
Nulliparous, *n*	128 (57.4%)
**Fetal**	
GA at first scan, weeks	22.3 (20–24.9)
EFW at first scan, g	487 (236–778)
Serial scan, times	5 (4–6)
GA at delivery, weeks	39.3 (35–41.1)
Birth weight, g	3,255 (2,409–3,688)
Boy: girl	120:103
**Outcome**	
Cesarean section, *n*	20 (9%)
Apgar score <7 at 1 min	6 (2.7%)
Apgar score <7 at 5 min	3 (1.3%)
NICU	3 (1.3%)
Neonatal mortality	0

*BMI, body mass index; NICU, neonatal intensive care unit*.

**Figure 3:**
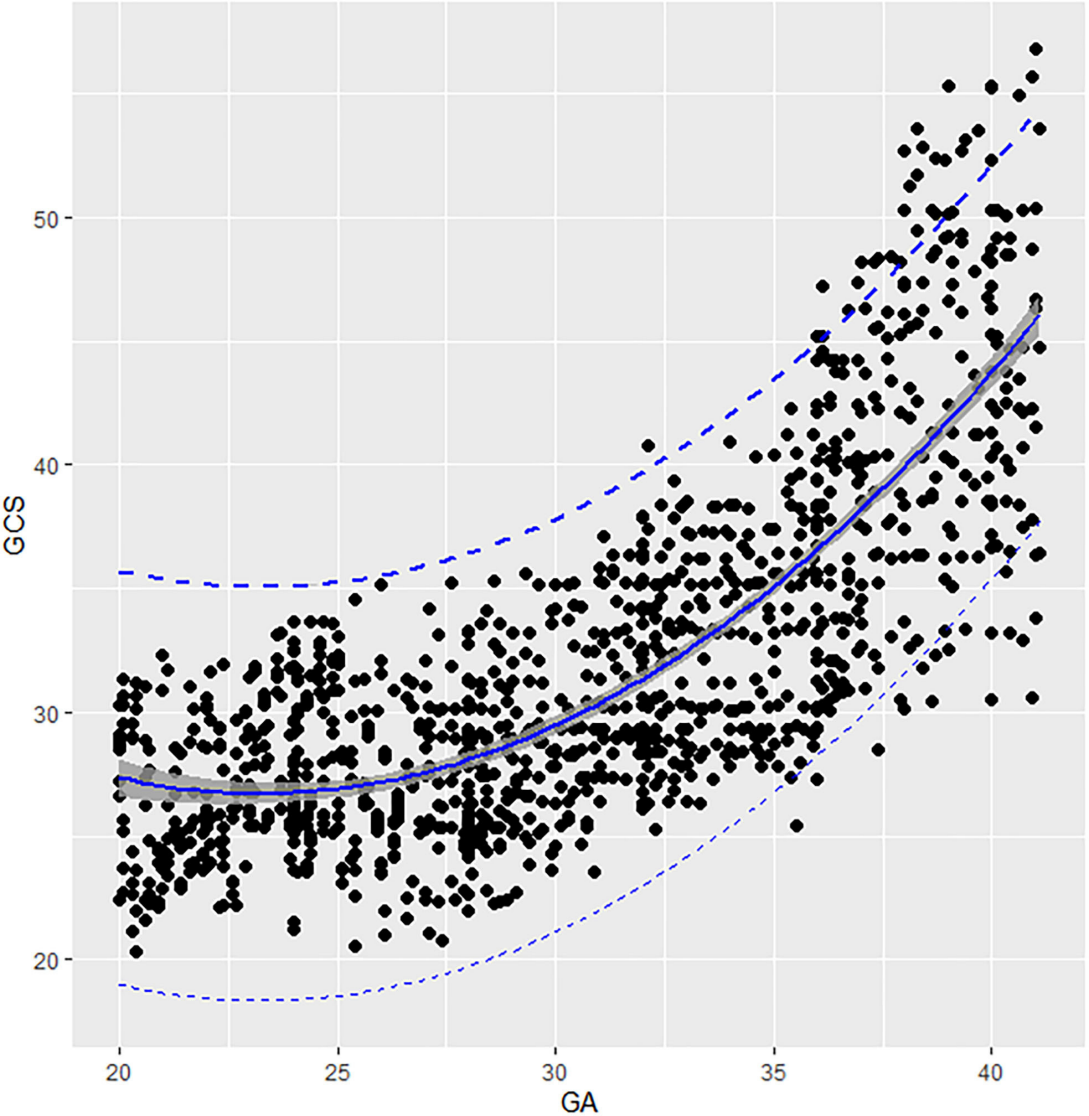
Scatter plots of the GCS based on GA: There was a second-degree fractional polynomial smoothing regression model between GCS and GA. Fitted percentiles according to gestational age are presented as solid lines. The 95% CI of the fitted mean value is presented as a gray area. The 5th and 95th percentile limits are presented as dashed lines. GA, gestational age; GCS, global circumferential strain.

The GCS of fetal AA presented a second-degree fractional polynomial smoothing regression along GA (*R*^2^ = 0.635, *P* < 0.05, Figure 3). Fetal aortic GCS remained unchanged at ~27.29% (20.36–35.6%) before 31 weeks and increased significantly from 31.36% (26.38–37.12%) at 31 weeks to 43.29% (30.5–56.78%) at term (*p* < 0.001). The 2.5th, 5th, 10th, 50th, 90th, 95th, and 97.5th percentiles for GA-specific ascending aorta GCS are listed in Table 2.

**Table 2:** Longitudinal reference percentiles of fetal ascending aorta global circumferential strain (GCS, %).

**GA (weeks)**	**Percentile**						
	**2.5th**	**5th**	**10th**	**50th**	**90th**	**95th**	**97.5th**
20	20.27	20.84	21.53	24.40	28.14	29.42	30.63
21	20.61	21.20	21.91	24.88	28.79	30.12	31.39
22	20.97	21.57	22.31	25.39	29.46	30.86	32.18
23	21.33	21.96	22.72	25.92	30.16	31.63	33.02
24	21.71	22.36	23.15	26.47	30.90	32.44	33.90
25	22.10	22.77	23.59	27.05	31.68	33.29	34.84
26	22.51	23.20	24.05	27.65	32.50	34.20	35.82
27	22.93	23.65	24.53	28.27	33.36	35.15	36.86
28	23.37	24.11	25.03	28.93	34.27	36.15	37.97
29	23.82	24.59	25.55	29.62	35.23	37.22	39.15
30	24.29	25.09	26.09	30.34	36.24	38.35	40.40
31	24.78	25.62	26.65	31.10	37.32	39.56	41.73
32	25.29	26.16	27.24	31.89	38.46	40.84	43.16
33	25.82	26.73	27.85	32.73	39.68	42.21	44.69
34	26.37	27.32	28.49	33.61	40.97	43.68	46.34
35	26.95	27.93	29.17	34.54	42.36	45.25	48.11
36	27.55	28.58	29.87	35.53	43.84	46.94	50.02
37	28.18	29.26	30.61	36.57	45.43	48.77	52.09
38	28.83	29.97	31.38	37.68	47.14	50.74	54.35
39	29.52	30.71	32.20	38.85	48.98	52.88	56.81
40	30.24	31.49	33.06	40.10	50.97	55.21	59.50
41	31.00	32.31	33.96	41.44	53.14	57.76	62.47

**Figure 4:**
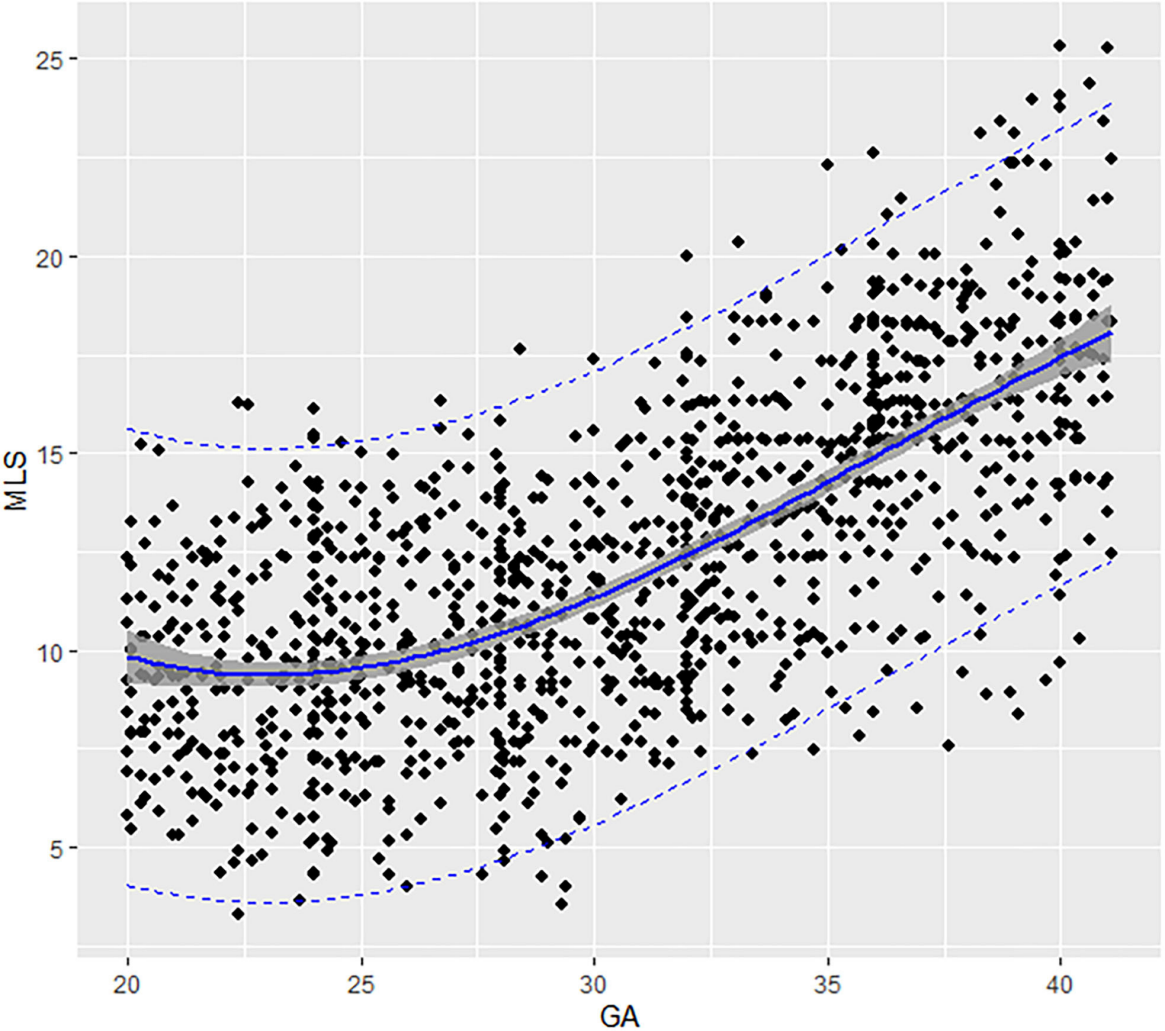
Scatter plots of the MLS based on GA: There was a third-degree fractional polynomial smoothing regression model between GCS and GA. Fitted percentiles according to gestational age are presented as solid lines. The 95% CI of the fitted mean value is presented as a gray area. The 5th and 95th percentile limits are presented as dashed lines. GA, gestational age; MLS, mean longitudinal strain.

Fetal aortic MLS presented a third-degree fractional polynomial smoothing regression along GA (*R*^2^ = 0.465, *P* < 0.05, Figure 4). The longitudinal strain of fetal AA remained steady at ~10.03% (3.28–17.62%) between 20 and 31 weeks and then increased significantly from 12.68% (7.42–20.1%) at 32 weeks to 17.5% (9.67–25.34%) at term (*p* < 0.001). The 2.5th, 5th, 10th, 50th, 90th, 95th, and 97.5th percentiles for GA-specific ascending aorta MLS are listed in Table 3.

**Table 3:** Longitudinal reference percentiles of fetal ascending aorta mean longitudinal strain (MLS, %).

**GA (weeks)**	**Percentile**						
	**2.5th**	**5th**	**10th**	**50th**	**90th**	**95th**	**97.5th**
20	3.69	4.26	4.95	7.83	11.37	12.47	13.50
21	3.94	4.53	5.24	8.19	11.80	12.92	13.97
22	4.20	4.80	5.53	8.55	12.24	13.38	14.45
23	4.46	5.08	5.83	8.93	12.68	13.85	14.93
24	4.73	5.38	6.14	9.31	13.14	14.32	15.43
25	5.01	5.67	6.46	9.70	13.60	14.81	15.93
26	5.30	5.98	6.79	10.10	14.07	15.30	16.44
27	5.60	6.29	7.12	10.51	14.55	15.80	16.95
28	5.90	6.62	7.47	10.92	15.04	16.30	17.48
29	6.21	6.95	7.82	11.35	15.53	16.82	18.01
30	6.53	7.29	8.17	11.78	16.03	17.34	18.55
31	6.86	7.63	8.54	12.22	16.55	17.87	19.10
32	7.20	7.99	8.92	12.66	17.07	18.41	19.66
33	7.54	8.35	9.30	13.12	17.59	18.96	20.22
34	7.90	8.72	9.69	13.58	18.13	19.51	20.80
35	8.26	9.10	10.09	14.05	18.67	20.08	21.38
36	8.62	9.48	10.49	14.53	19.22	20.65	21.97
37	9.00	9.88	10.91	15.02	19.78	21.23	22.57
38	9.38	10.28	11.33	15.51	20.35	21.82	23.17
39	9.78	10.69	11.76	16.02	20.93	22.41	23.79
40	10.18	11.11	12.20	16.53	21.51	23.02	24.41
41	10.59	11.54	12.65	17.05	22.10	23.63	25.04

The paired-samples *t*-test demonstrated that GCS was significantly higher than MLS in the ascending aorta wall during the fetal period (*p* < 0.001).

**Figure 5:**
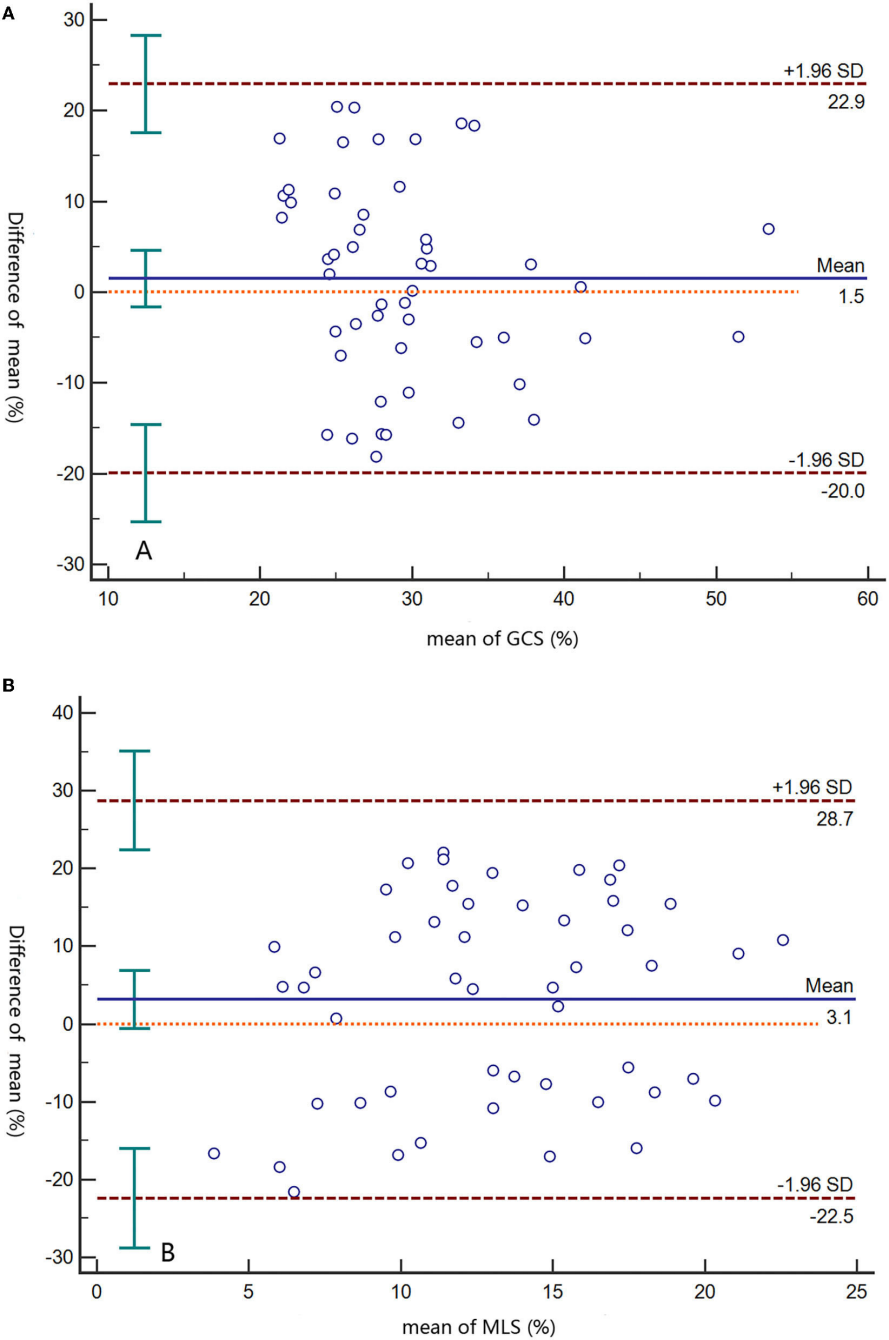
Bland–Altman plots of interobserver differences at the GCS **(A)** and MLS **(B)**. Solid lines represent the means, and dashed lines indicate limits of agreement. GCS, global circumferential strain; MLS, mean longitudinal strain.

The interobserver differences for GCS and MLS were 1.5% (95% limit −20–22.9%) and 3.1% (95% limit −22.5–28.7%), respectively (Figure 5). The intra-observer differences for GCS and MLS were 0.1% (95% limit −11.2–11.5%) and −1.7% (95% limit −19.9–16.6%), respectively (Supplementary Figure 2).

## Discussion

To our knowledge, this study first assessed the circumferential and longitudinal strain of the fetal ascending aortic wall and demonstrated obviously greater circumferential strain than longitudinal strain in the fetal AA wall. Both strains remained steady before the late trimester and then gradually increased until delivery.

Fetal aortic GCS remained unchanged before 31 weeks and increased significantly from 31.36% (26.38–37.12%) at 31 weeks to 43.29% (30.50–56.78%) at term. Similarly, MLS remained steady between 20 and 31 weeks and then increased significantly from 12.68% (7.42–20.1%) at 32 weeks to 17.5% (9.67–25.34%) at term. Both circumferential and longitudinal staining of fetal AA presented steady before the late trimester and then gradually increased until delivery. This unique growth trajectory of human fetal ascending aorta elastic properties was in agreement with the change in the elastin/collagen ratio in aortic wall development. The aorta's elastic behavior depends largely on the amount and organization of elastic fibers and collagen fibers in the wall (4). Berry et al. (3) sequentially observed the scleroprotein content of the human aorta and found that there was a different growth curve of increased elastin and collagen during human aortic development. Collagen increased rapidly from 27 to 37% of dried fat-free tissue (DFFT) between GA 12 and 30 weeks, then increased slowly to 41% of DFFT util term and remained constant thereafter. Elastin increased DFFT from 13 to 24% between GA 14 and 35 weeks and then increased dramatically to 42% at 3 months postnatal. These different increase rates of collagen and elastin during aortic development result in the elastin/collagen ratio remaining fairly constant before the late trimester and a dramatic increase until postnatal 3 months.

The fetal aortic elastic property development model in our results was inconsistent with the previous limit reports that focus on other wall elastic parameters, i.e., the aortic diameter change (18) and PWV (19). Mori et al. (18) measured the serial systolic and diastolic diameters of fetal descending aortae in 22 normal pregnancies and then calculated the diameter difference (pulse amplitude). They found that the pulse amplitude increased linearly with gestational ages from 28 to 40 weeks and suggested linear growth of aortic distensibility. Miyashita et al. (19) cross-sectionally measured descending aortic PWV in 65 normal fetuses and showed stable PWVs at ~2 m/s during the fetal period, suggesting unchanged and relatively lower arterial stiffness in the fetal aortic wall than in postnatal life. In addition to various sample sizes and aortic segments of interest, distinct evaluation techniques were the main factor contributing to the discrepancy between our results and the abovementioned reports. GCS and MLS in our study, computed from the two-dimensional speckle tracking technique VVI, calculate the displacement of the speckles (20) in relation to each other within the traced aortic wall in circumferential and longitudinal directions separately and therefore directly represent the elastic stretch extent under force in theory. Kim et al. (15) observed 137 adult descending abdominal aorta circumferential stains using VVI and showed that aortic strain was significantly negatively associated with both age and the parameters of arterial stiffness measured by PWVs. Histologic and biomolecular data also proved that strains derived from the VVI technique negatively correlated with collagen content in human aortic wall tissue (21) and elastin soluble fragment amount in plasma (22). Together, VVI has the potential benefit for directly evaluating aortic wall mechanic elastic properties under cyclic heart force.

Our study observed a higher GCS than MLS in the fetal ascending aortic wall, suggesting greater strain on the circumferential orientation than on the longitudinal orientation of the AA wall. As the most proximal segment of the aorta, the ascending aorta is subjected to the largest cyclic circumferential stress and axial stress from the blood pressure and motion of the heart, respectively (23). These stresses definitely cause wall displacement (strain), and *in vivo* experiments have demonstrated a non-linear positive stress-strain relationship in human arteries (24). The more dominant aortic circumferential strain in our study can be interpreted by the unique microstructure of the aorta. The healthy human arterial wall is divided into three layers: intima, media, and adventitia. The intima is composed mainly of a single layer of endothelial cells, a thin basal membrane, and a subendothelial layer of collagen. The media, the largest and middle layer, is composed of smooth muscle cells, a network of elastic and collagen fibrils, and elastic laminae. Elastic fibers and collagen interconnect the elastic laminae, forming a continuous three-dimensional helical network in which the fibers are oriented in a near-circumferential direction (25). The adventitia is composed of thick bundles of collagen fibrils that are also arranged helically (26). Such a helical structure with most fibers oriented circumferentially in the aortic wall may be designed to bear the predominant circumferential strain.

This study has several limitations. First, we did not enroll fetuses <20 weeks because the second-trimester ultrasound scan mainly starts at 20 weeks in the region of China. However, the results in our study were statistically sufficient to demonstrate the development mode of elasticity in the human fetal ascending aortic wall based on longitudinal observations. Second, the relatively low frame rate in the VVI technique could undermine the accuracy evaluation of wall deformations. We adapted the reduction of scanning area and optimization of the focus position and depth to increase the frame rate and showed good performance on the interobserver agreement.

## Conclusion

The present study demonstrated that fetal aortic GCS and MLS can be easily measured with good interobserver agreement using the VVI technique. Both circumferential and longitudinal aortic strains remained steady before the late trimester and then gradually increased until delivery. The 2.5th, 5th, 10th, 50th, 90th, 95th, and 97.5th percentiles for GA-specific ascending aorta GCS and MLS were provided to help further study pathologic conditions.

## Data Availability Statement

The original contributions presented in the study are included in the article/Supplementary Material, further inquiries can be directed to the corresponding author/s.

## Ethics Statement

The studies involving human participants were reviewed and approved by the Institutional Review Board at the Second Xiangya Hospital of Central South University. The patients/participants provided their written informed consent to participate in this study.

## Author Contributions

SZ and RX contributed to conception and design of the study. XZ, YL, and SZ organized the database. YL and DZ performed the statistical analysis. RX wrote the first draft of the manuscript. SZ, ML, and JZ wrote sections of the manuscript. All authors contributed to manuscript revision, read, and approved the submitted version.

## Funding

This study was supported by the State Natural Sciences Foundation of China (Grant No. 81871372) and Natural Science Foundation of Hunan Province (Grant No. 2019JJ40425). The funding body was not involved in the design of the study; the collection, analysis, and interpretation of the data; or the writing of the manuscript.

## Conflict of Interest

The authors declare that the research was conducted in the absence of any commercial or financial relationships that could be construed as a potential conflict of interest.

## Publisher's Note

All claims expressed in this article are solely those of the authors and do not necessarily represent those of their affiliated organizations, or those of the publisher, the editors and the reviewers. Any product that may be evaluated in this article, or claim that may be made by its manufacturer, is not guaranteed or endorsed by the publisher.
