# Supplementary figures and images for: Maturation Fetus Ascending Aorta Elastic Properties: Circumferential Strain and Longitudinal Strain by Velocity Vector Imaging
Source: Front Cardiovasc Med. 2022 Feb 28;9:840494. doi: 10.3389/fcvm.2022.840494 (PMC8918822; doi:10.3389/fcvm.2022.840494)

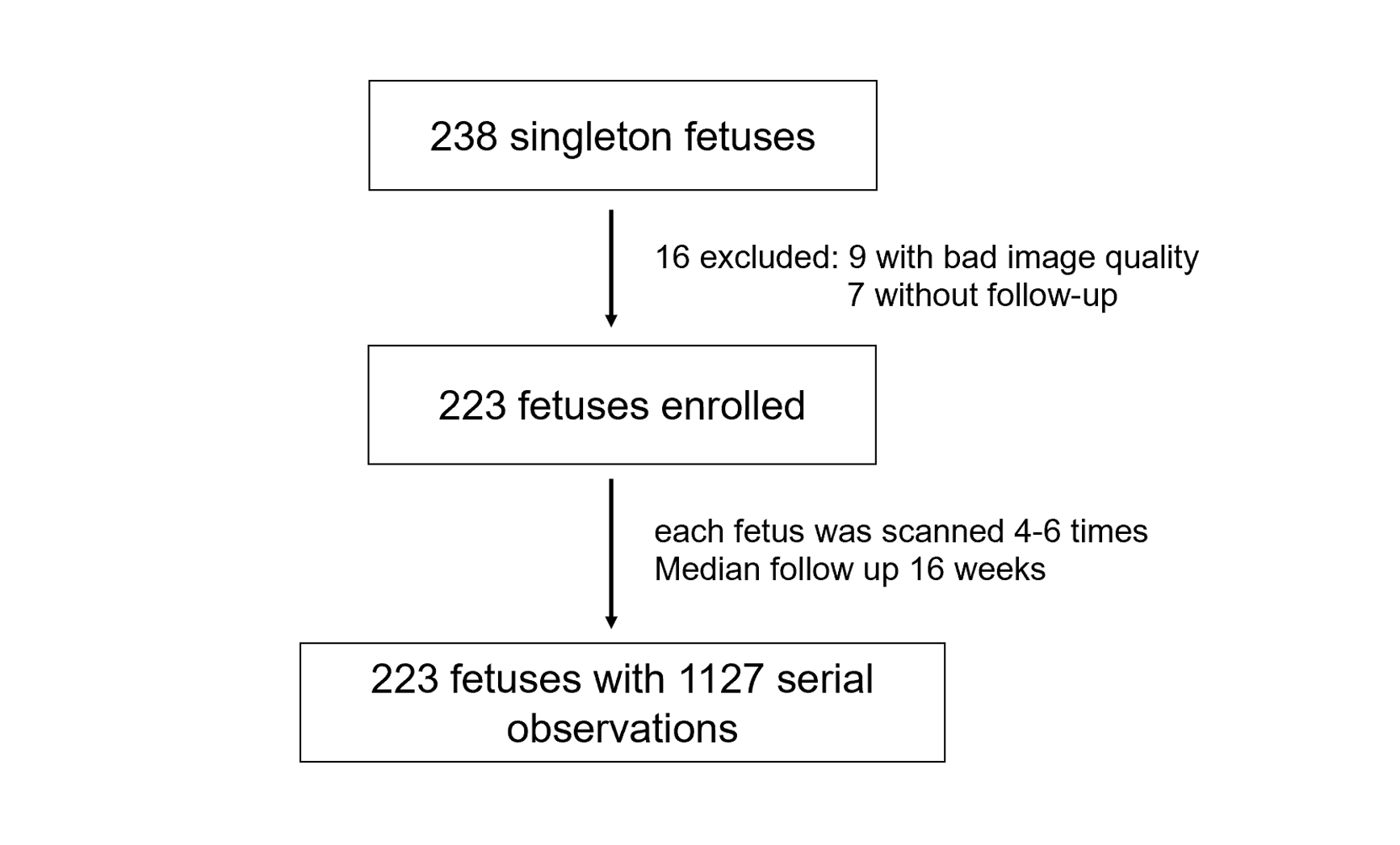

Supplement: Supplementary file 1 [file Image_1.TIF]

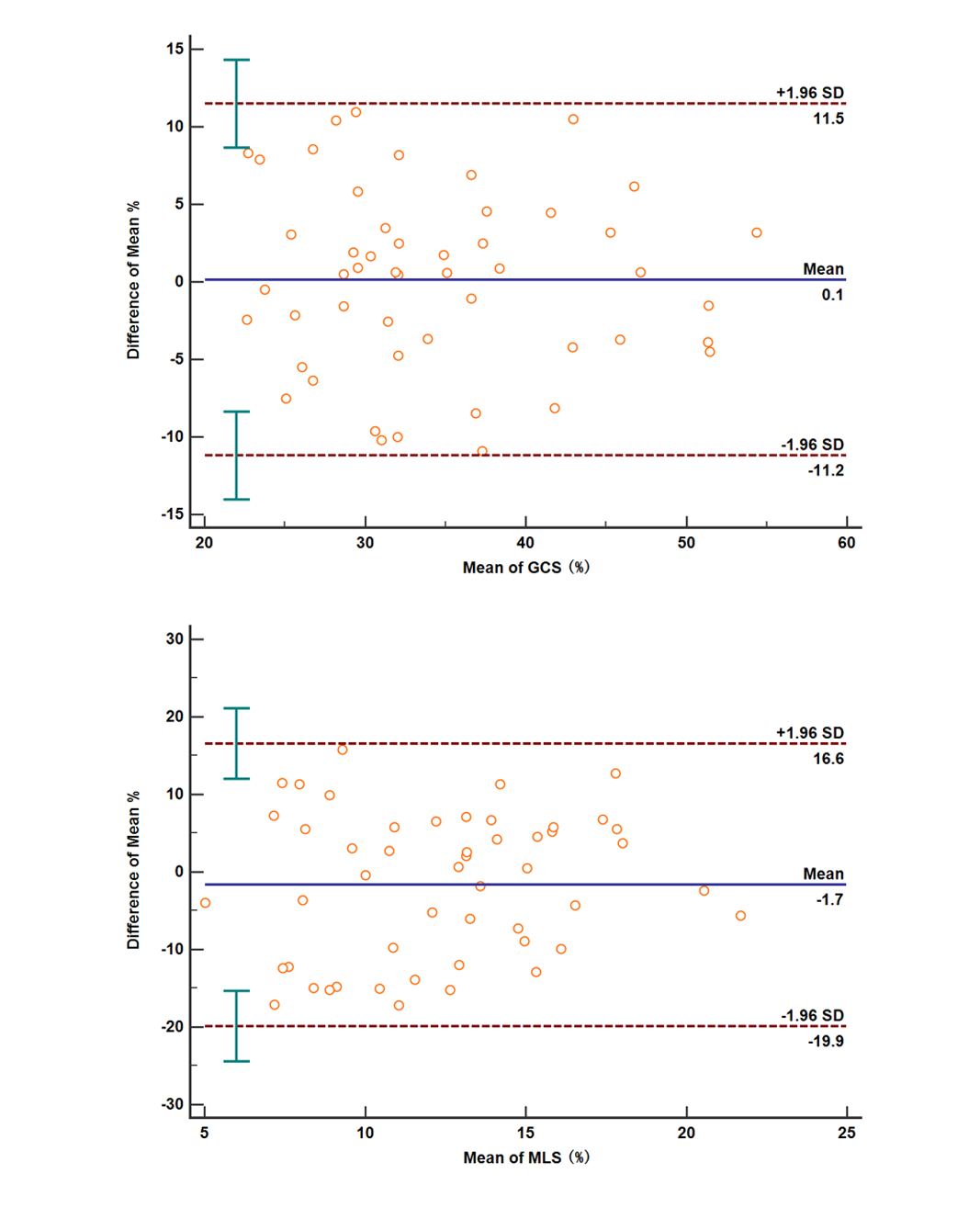

Supplement: Supplementary file 2 [file Image_2.TIF]
